# Survival and Predictors of Mortality among Adults Initiating Highly Active Antiretroviral Therapy in Ethiopia: A Retrospective Cohort Study (2007-2019)

**DOI:** 10.1155/2022/5884845

**Published:** 2022-11-23

**Authors:** Yimam Getaneh, Feng Ning, Qianxin He, Abdur Rashid, Desta Kassa, Yibeltal Assefa, Feng Yi, Lingjie Liao, Yiming Shao

**Affiliations:** ^1^State Key Laboratory for Diagnosis and Treatment of Infectious Diseases, National Clinical Research Center for Infectious Diseases, Collaborative Innovation Center for Diagnosis and Treatment of Infectious Diseases, The First Affiliated Hospital, College of Medicine, Zhejiang University, Hangzhou, China; ^2^Ethiopian Public Health Institute, Addis Ababa, Ethiopia; ^3^State Key Laboratory for Infectious Disease Prevention and Control, National Center for AIDS/STD Control and Prevention, Chinese Center for Disease Control and Prevention, Beijing 102206, China; ^4^School of Medicine, Nankai University, Tianjin, China; ^5^University of Queensland, School of Public Health, Queensland, Australia; ^6^Changping Laboratory, Beijing, China

## Abstract

**Background:**

Studies have shown high early mortality after initiation of highly active antiretroviral therapy (HAART). We examined change in three-year survival and predictors of mortality of patients initiating HAART in Ethiopia since 2007 to 2019.

**Methods:**

A retrospective cohort study was conducted in 47 health facilities (HFs) using records of 11,013 adult patients initiating HAART from 2007 to 2019. Study subjects were stratified as four different cohorts based on their calendar year of HAART initiation: 2007-2010, 2011-2013, 2014-2016, and 2017-2019. HFs were selected using probability proportional to size of patients. Survival rate and predictors of mortality were estimated by the calendar year using the Kaplan-Meier and Cox proportional hazard, respectively. We generated a pooled estimate of survival rate and predicators of mortality.

**Results:**

Data from 1881, 3868, 3004, and 2260 patients were retrieved from each of the cohorts. Overall mortality for all cohorts at all times was 10.3%. A gradual decline of mortality was observed in the first three years of follow-up since 2007-2016 which were 21.37%, 10.03%, and 4.34% among patients who initiated HAART in 2007, 2011, and 2014 respectively. A mortality jump of 9.25% was observed among patents initiating HAART in 2017, which coincided with political instability happened in the country. Of the 21,638 person-years of follow-up among 11,013 adults, mortality was 5.23/100 person-years, while disaggregated by the cohorts, it was 14.77, 5.06, 2.12, and 4.17 per 100 person-years, respectively. Among all the cohorts, patients with CD4 count of ≤200 cells/mm^3^, unsuppressed viral load, poor adherence, and drug resistance in all cohorts, respectively, have overall 2.0 (95%CI = 1.35 − 2.69), 4.66 (95%CI = 2.53 − 6.72), 6.78 (95%CI = 3.4 − 10.3), and 10.02 (95%CI = 6.91 − 13.82) times of mortality risk than those without. Patients with bedridden for cohort initiating HAART during 2007 and 2011 were 2.0 (95%CI = 1.35 − 2.69) times of mortality risk than those without.

**Conclusion:**

Patients initiating HAART from 2007 to 2016 have continuously improved their survival during three-year cohort follow-up in Ethiopia. The significant decline of survival among those who initiate HAART as of 2017 calls for program intervention. Low CD4 counts, unsuppressed viral load, poor adherence, and drug resistance could be used as predictors for increased mortality to monitor the quality of HAART and improve clinical management of HIV/AIDS patients.

## 1. Background

Human immunodeficiency virus (HIV) is a global public health challenge and main cause for morbidity and mortality in sub-Saharan African countries since the first case was reported in the 1980s [1]. Expanded access to highly active antiretroviral therapy (HAART) significantly reduced morbidity and mortality due to HIV infection [2]. The aim of HAART is to significantly reduce viral load to undetectable levels (up to 50 copies/ml) resulting to slow disease progression by increasing CD4 cell count [3]. As one of the benefits of HAART, there was a 26% decline in AIDS-related deaths globally since 2010. Low survival and high mortality at initial HAART stage is a critical challenge to the local public health programs in the high HIV/AIDS-burden countries [4].

HIV/AIDS-related mortality of patients may depend on multiple general features including host factors, the patterns of diseases present, access to health care, diagnostic routines, and therapeutic interventions at the local level [5–7]. Specific risk factors related to mortality include higher viral load, low CD4 count, regimen intolerance, previous exposure to ARV drugs, malnutrition, medication adherence, missed clinic appointment, TB-HIV coinfection, delayed ART initiation, and age group [3, 8–10].

In Ethiopia with an estimated national prevalence of 0.96% and a total of 622,326 people living with HIV (PLHIV) in 2020 [11], free ART service was launched in hospitals by 2005 and primary healthcare centers in 2006 as a part of the service scaling-up [12]. Since 2015, Ethiopia adopted the global recommendation of test and treat strategy [13]. Despite the few studies that addressed survival among people taking HAART in Ethiopia, there has been inconsistency with the reports. These studies indicated survival among PLHIV taking HAART in Ethiopia ranging from 4.2% to 43% during the 36 to 72 months of follow-up, in different settings [3, 10, 14–19]. It has been also revealed that mortality rates of HIV patients were high within the first year of ART treatment in Ethiopia, and around 20,000 PLHIV died each year [20–23].

Robust evidence on survival rate and predictors of mortality at early stage of HAART initiation is, therefore, the crucial information needed for evidence-based decision-making in order to improve the patients' clinical management. In this regard, previous studies in Ethiopia were inconclusive. Therefore, this study was undertaken with objectives to estimate survival rate and identify predictors of mortality among PLHIV during the first three years of HAART between the years 2007 and 2019.

## 2. Methods

### 2.1. Study Design and Setting

A retrospective cohort study was conducted from December 2019 to March 2020. During this time of data collection, we retrieved data from 11,013 study participants attending 47 health facilities (HFs) who were initiated for HAART as of 2007. There were 9 regional and 2 city administrations and 1,047 HFs providing ART service for 578,739 PLHIV by 2019 in Ethiopia [24].

### 2.2. Study Population and Eligibility

All HIV-infected adults (age ≥ 15 years old) who were enrolled for ART care and support from December 2007 to November 2019 in the 47 selected HFs were considered as a study population.

### 2.3. Sample Size Determination and Sampling Procedures

We used a two-stage, stratified cluster sampling for this study. First, we include HFs which was providing ART service on or before 2007 (*N* = 311). For feasibility reason, we selected 47 (15%) HFs using probability proportional to population size (PPPS) of HAART patients in the HFs. In the second stage, we stratified the study participants in the selected HFs in a different cohort groups 2007-2009, 2010-2013, 2014-2016, and 2017-2019 based on the calendar year of HAART initiation. The list of ART patients with their unique ART number was retrieved from the ART clinic of the HFs, and a sample of ART patients was selected from each of the selected HFs using systematic random sampling according to the existing list of ART patients.

The sample size was determined for each of the cohorts by using EPI Info version 7.2.1 [25]. For estimating the sample size, We assum type I error to be 5% with 80% power using two population proportions on exposure status. Two populations were categorized by CD4 count as the main exposure variable for HIV/AIDS-related deaths during the follow-up period. The assumed proportions of probability of survival of adults whose CD4 count ≤ 200 cells/mm^3^ were 35.5% during 2007-2009 [17], 59% during 2010-2013 [26], and 66.4% during 2010-2019 [27].

With a design effect of 1.5, the sample sizes for each of the cohorts (2007-2009, 2010-2013, 2014-2016, and 2017-2019) were 1730, 3558, 2114, and 2114, respectively. Hence, the minimum sample size required for this study was calculated to be 10,131. Assuming exclusion of records of lost to follow-up (LTFU), transfer-in, and transfer-out, patients initiated for HAART before 2007 and children under the age of 15 to be 23% the final sample size required were 12,462.

### 2.4. Study Variables

#### 2.4.1. Independent Variables

The independent variables are baseline demographic characteristics (age, gender, marital status, educational status, and occupation) and clinical characteristics (World Health Organization (WHO) clinical stage, CD4 count (cells/mm^3^), hemoglobin (mg/dl), ART regimens, adherence, and baseline functional status (ambulatory or bedridden)).

#### 2.4.2. Dependent Variable

Functional status at 3 years of follow-up (alive or death) were considered as outcome variable.

Baseline functional status was categorized into two groups: normal activity and bedridden (>50% of the day during the past month) [28] as recorded by the ART site physician. The WHO clinical stage was categorized into four groups (stage I, stage II, stage III, and stage IV) [29]. CD4 count was categorized as ≤200 cells/mm^3^ and >200 cells/mm^3^ as defined by WHO consolidated guideline [30]. Medication adherence was measured using self-report measure consisted of a single item querying the number of prescribed doses the participant had missed in a specified time period (*n* = 30 days) [31]. Hence, classification was defined as poor if the percentage of the missed dose was <85% (>6 of the 30 doses or >9 of the 60 doses) as documented by ART physician, fair adherence was defined if the percentage of the missed dose was between 85% and 94% (3-5 of the 30 doses or 3-9 of the 60 doses), and good adherence was defined if the percentage of missed dose is above >95% (<2 of the 30 doses or <3 of the 60 doses). Hemoglobin reference range were defined as ≤12.5 mg/dl or >12.5 mg/dl as defined by the reference range for the normal adult population in Ethiopia [32].

Date of death was recorded either electronically or from the medical register of HIV-infected patients who died during the follow-up period from all causes. Patients missing their follow-up visits according to their appointment schedule for more than 3 months were recorded as loss to follow-up for whom the date of the last registered follow-up visit was recorded to define as loss to follow-up.

### 2.5. Measurement of Plasma Viral Load and CD4 Count

Plasma viral load (PVL) test was performed at the regional testing laboratories in Ethiopia, using two different approaches: (1) COBAS® AmpliPrep/COBAS TaqMan® HIV-1 version 2.0 (v2.0), based on in vitro amplification of HIV-1 RNA from plasma with detection thresholds ranging from 20 (lower) to 10,000,000 (upper) RNA copies/ml designed specifically for HIV-1 groups M and O, and (2) Abbott Real-Time HIV-1 test Ref 2G3190, based on in vitro amplification by RT-PCR for the quantification of HIV-1 in plasma. Both rounds of VL tests were performed as per manufacturers' instructions. Second viral load tests were done at six-month follow-up for patients with initial viral load ≥ 1,000 copies/ml using similar techniques.

CD4 cells were enumerated using whole blood samples prior to extraction of the plasma samples using FACScount® automated cell counter (Becton-Dickinson, Franklin Lakes, New Jersey, USA) at the health facilities.

### 2.6. RT-PCR and Nested PCR

In-house assay protocols developed and validated by the Chinese Center for Disease Prevention and Control (CCDC) were used for PCR amplification and sequencing [33]. Briefly, one-step RT-PCR was performed using 50 ml reaction, which consisted of 10 ml of RNA extracts, 0.16 mM each of primers PRTMF1 and RT-R1, and 0.5 ml SuperScript™ III One-Step RT/Platinum Taq HiFi Enzyme Mix and 1x reaction buffer mixture containing Mg_2_^+^ and deoxyribonucleotide triphosphates (dNTPs) (Invitrogen, Carlsbad, CA). RT-PCR condition was an initial cycle RT step at 50°C for 45 min and 94°C for 2 minutes and followed by 40 cycles of PCR at 94°C for 15 sec, 50°C for 20 sec, and 72°C for 2 minutes and an extension at 72°C for 10 min. For nested PCR, 2 ml of RT-PCR product was added to a 50 ml reaction containing 0.12 mM of the inner primers PRT-F2 and RTR2, 1x GeneAmp Gold Buffer II, 2 mM MgCl_2_, 400 mM each dNTP with 2.5 U of AmpliTaq Gold LD DNA polymerase (Applied Biosystems, Foster City, CA). After initial denaturation at 94°C for 4 min, 40 cycles of PCR were performed in GeneAmp 9700 thermocycler with the PCR conditions as 94°C for 15 sec, 55°C for twenty seconds, and 72 for 2 min and following an extension at 72°C for 10 min. A 1% agarose gel electrophoresis with a product size of 1,084 base pairs was used to confirm nested PCR product. The confirmed PCR products were purified using Qiagen PCR purification kits and used for cycle sequencing reaction with BigDye terminator cycle sequencing kit 3.1 (Applied Biosystems, CA).

### 2.7. Data Collection and Processing

Data were collected from standard medical record registers and electronic records at ART sites adopted by the Ministry of Health of Ethiopia. There were three available registers: the first register was the Pre-ART register where all confirmed HIV-positive patients who visit ART site were registered. Then, all the patients who started ART regimen were transferred to ART register at the date of treatment initiation. The third register was patient's follow-up form. For each patient, the initial follow-up visit was scheduled for every two weeks after treatment initiation, and then on a monthly basis and as required based on adherence of the patient, medical records were updated for every patient during each follow-up visit. There was electronic medical record which comprises all history of HIV-positive clients since identified positive.

The data elements retrieved include clinical characteristics and laboratory results and WHO clinical stage, CD4 counts, hemoglobin, ART regimens, medications, and functional status (alive or dead). Moreover, data of demographic characteristics (age, gender, marriage status, educational status, and occupation) and individual factors (medication adherence) were also retrieved. Data of LTFU was separately retrieved and analyzed from the recorded report.

Data management training was given for data collectors and supervisors who were health professionals working at the selected health facilities. The procedure was controlled by the investigator of the study. Completed data collection tools examined their completeness and consistency. Data from the records were entered in to a preestablished data entry template (i.e., CSPRO) using tablet computers.

### 2.8. Statistical Analysis

The patients' characteristics were described in terms of frequency, mean/median value for continuous data, and percentages for categorical data. Continuous variables were also compared using *t* tests or rank sum tests after testing the normality of the distribution using the Shapiro-Wilk test. HAART initiation date and the date of outcome (alive or death) were used as the start as well as end point of follow-up time, respectively. Patients who were alive and in care were censored as of the date of their last clinic visit.

Data analysis was done for each of the cohorts according to their calendar year. Follow-up time (in year) was calculated from the date of ART initiation to the date of death or censoring. The Kaplan-Meier analysis was done to estimate the survival rate of the study participants. The Cox proportional hazard regression was calculated to determine significant factors related to survival rate. We used both crude and adjusted hazard ratios (HRs) at 95% confidence intervals, and candidate variables with *P* < 0.2 were considered to the multivariable Cox regression model. Independent variables with values of <0.05 within the multivariable Cox regression model were considered statistically significant predictors of mortality. The proportional hazard assumption was checked by both graphical and statistical tests of the goodness-of-fit test (Schoenfeld's method). All data analysis was done using STATA version 16.0 for each of the cohorts.

We finally conducted a meta-analysis for the survival rate of the different cohorts to get a pooled point estimate. Moreover, we conducted a meta-analysis for the predictors of mortality also to generate the pooled estimate of hazard ratio for the duration of 2007-2019.

## 3. Result

### 3.1. Characteristics of the Study Participants

A total of 11,013 HIV patients attending 47 selected HFs were included in this study. Briefly, a data of 1881, 3868, 3004, and 2260 patients were retrieved from the cohorts.

Of the total patients, 7152 (64.94%) were female and 10,089 (91.61%) were from the urban setting. The mean age of the cohorts was 40 years, ranging from 15 to 90 years old. More than half of the study participants were from Amhara, Oromia, Tigray, and SNNPR areas, which accounted 1555 (14.1%), 1542 (14%), 1504 (13.6%), and 1392 (12.6%), respectively. More than two-thirds of study participants were either with primary school education (42.9%) or no formal education (25%). About half of the study participants 5381 (48.9%) were married while 2261 (20.5%) of them were divorced, and equal number of them were widowed 2087 (19%). Predominant population of the study was government employees, house wives, and merchant which accounted 2087 (19%), 1840 (16.7), and 1767 (16%), respectively (Table 1).

### 3.2. Mortality among People Initiating HAART in Ethiopia

**Figure 1:**
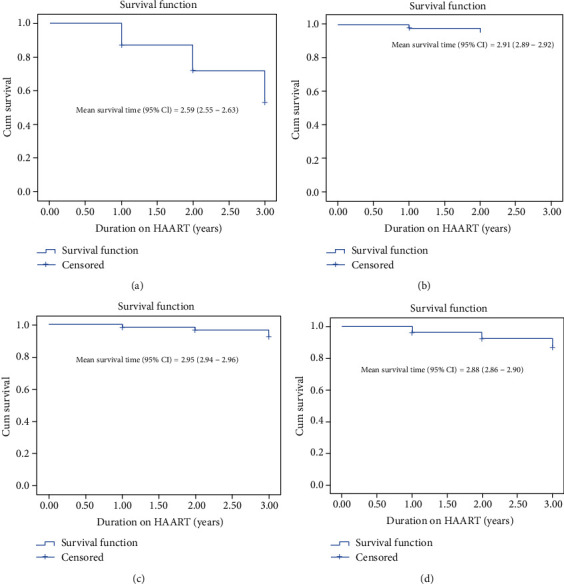
Overall survival among people initiating HAART in Ethiopia. (a) Survival among cohort 2007-2010. (b) Survival among cohort 2011-2013. (c) Survival among cohort 2014-2016. (d) Survival among cohorts 2017-2019.

**Figure 2:**
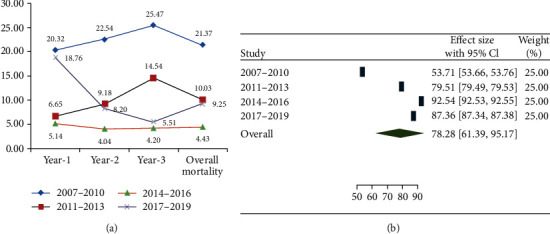
(a) Proportion of mortality among people initiating HAART in Ethiopia by different calendar year of HAART initiation (A, B, C, and D). (b) Pooled estimate for overall survival among people initiating HAART in Ethiopia (2007-2019).

Of the 21,638 person-years of follow-up among 11,013 study participants, mortality was 5.23/100 person-years, while disaggregated by the cohorts, it was 14.77, 5.06, 2.12, and 4.17 per 100 person-years, respectively. Mortality among people initiating HAART in Ethiopia during the year 2007 to 2019 at the first three years of follow-up was 10.3%. For all the cohorts, overall mortality was 21.37%, 10.03%, 4.34%, and 9.25%, respectively (Figure 1 and Table 2). Patients in the cohort initiating HAART by 2007 had the highest mortality accounting 20.32% at the first year followed by 22.54% and 25.47% during the second and third year of follow-up, respectively. For cohort initiating HAART by 2010, mortality was 6.65%, 9.18% and 14.54% during the first, second and third year of the follow-up period, respectively. Moreover, mortality for cohort initiating HAART by 2014 was 5.14%, 4.04%, and 4.20% during the first, second, and third year follow-up periods, respectively. However, there were a relatively higher mortality during the cohorts of initiating HAART by 2017 compared to 2014 which accounted 18.76%, 8.2%, and 5.51% during the first, second, and third year of follow-up, respectively (Figure 2).

While mortality was disaggregated by the different demographic and clinical characteristics, the highest mortality for the cohort were among patients initiating HAART by 2007 were from Gambella and Amhara regions which was 25% and 24.47%, respectively. For the cohort initiating HAART in 2010, the high mortality was from Dire Dawa and Addis Ababa which accounted 12.73% and 11.98%. Considering cohort initiating HAART by 2010, there were no difference in mortality among the different regions in the country while the high mortality of cohort initiating HAART by 2017 was from Harari and Dire Dawa which accounted 14.69% and 14.63%, respectively. There were no differences among the cohorts with regard to the different age bands, gender, residency, and educational status. However, the high mortality was among divorced and widowed during the cohorts initiating HAART by 2007 which accounted 23.10% and 21.48%, respectively. Drivers were at higher mortality compared to other occupations across all the cohorts since 2007 to 2019 which accounted 29.87% during cohorts initiating HAART by 2007 to 11.86% by cohorts initiating HAART by 2017 (Table 2).

The highest mortality during the cohort's initiating HAART in 2007 was among people taking AZT, 3TC, and NVP which accounted 50%; then after, there were similarities in mortality among the different regimens by cohorts initiating HAART by 2010 and 2014 cohorts while AZT, 3TC, and EFV accounted the highest mortality by the cohorts initiating HAART by 2017 which accounted 17.50%. The high mortality was among bedridden patients by the cohorts of initiating HAART by 2007 which accounted 22.01%, and there were relative similarities among the different cohorts thereafter. Mortality among patients with poor adherence was high across all the cohorts which accounted 90.91%, 84.21%, 50.00%, and 64.01%, respectively. There was high mortality among patients with CD4 count ≤ 200 cells/mm^3^ across all the different segments of the cohorts which accounted 25.73%, 12.55%, 5.65%, and 18.65% during all the cohorts, respectively (Table 2).

### 3.3. Survival among People Initiating HAAR T in Ethiopia

Overall survival rate among PLHIV initiating HAART in Ethiopia from 2007 to 2019 within three years of follow-up was 78.8% (95%CI = 61.39 − 95.17) (Figure 1). Survival among all the cohorts was found to be 53.71% (95%CI = 53.66 − 53.76), 79.51% (95%CI = 79.49 − 79.53), 92.54% (95%CI = 92.53 − 92.55), and 87.36% (95%CI = 87.34 − 87.38), respectively (Figure 1). The mean survival time among all the cohorts was 2.59 (95%CI = 2.55, 2.63), 2.90 (95%CI = 2.89, 2.92), 2.95 (95%CI = 2.94, 2.96), and 2.88 (95%CI = 2.87, 2.90), respectively (Figure 1).

### 3.4. Predictors of Mortality among People Taking HAART in Ethiopia

Adherence, CD4 count, and functional status were found to be predictors of mortality among people taking HAART in Ethiopia. Briefly, patients with poor adherence were at 4.65 (95%CI = 2.90, 7.48), 12.29 (95%CI = 7.06, 21.40), 8.54 (95%CI = 2.71, 26.94), and 7.07 (95%CI = 3.74, 13.38) times of mortality risk for all cohorts, respectively, compared to those with good adherence. CD4 count of ≤200 cells/mm^3^ was at 2.71 (95%CI = 2.01, 3.66), 2.57 (95%CI = 1.93, 3.42), 2.46 (95%CI = 1.60, 3.77), and 2.70 (95%CI = 2, 3.64) times of mortality risk for all the cohorts, respectively, compared to those with CD4 count > 200 cells/mm^3^. Being bedridden were at 1.92 (95%CI = 1.28, 2.89) and 2.30 (95%CI = 1.40, 3.78) times risk of mortality among the cohorts of initiating HAART by 2007 and 2010, respectively (Table 3).

From our pooled estimate result, patients with poor adherence were at 6.78 (95%CI = 3.4 − 10.3) times risk of death compared to those who had good adherence. Patients with CD4 count of ≤200 cells/mm^3^ were at 2.6 (95%CI = 2.2 − 3.0) times risk of death compared to those with CD4 count greater than 200 cells/mm^3^. Bedridden patients were at 2.0 (95%CI = 1.35 − 2.69) times risk of death compared to ambulatory patients. Being drug resistant for HIV was found to be a 10-fold risk of mortality compared to those who were susceptible 10.02 (95% CI: 6.91, 13.82). Moreover, patients who were not virally suppressed at baseline were at 4.66 times risk of death (95%CI = 2.53, 6.72) (Table 3).

## 4. Discussion

Mortality among people initiating HAART in Ethiopia during the year 2007 to 2019 in the first 3 years since ART initiation was 10.3%. Mortality significantly varied by the follow-up year 2007-2009, 2010-2013, 2014-2016, and 2017-2019 which was 21.37%, 10.03%, 4.34%, and 9.25%, respectively. The cohort initiating HAART by 2017, who was the first group to initiate HAART during 2007-2009, accounted for the highest rate of mortality of 21.37%. This was not consistent with other study conducted in Ethiopia during the year 2006-2010 that reported 7% mortality at 36 months of follow-up [34]. Other study conducted in Gonder, Ethiopia, in the year 2010-2014 reported mortality at 60 months of follow-up to be 13.67% [8] which is relatively similar to our second HAART cohort, initiating HAART during 2010-2013, that revealed a 10.03% mortality. Despite the study group were children, a study conducted in Gamo Gofa Zone, Ethiopia, conducted from 2009 to 2016 reported mortality in the 60 months of follow-up to be 26.1% which was not in line with our report that showed 4.34% mortality in the year 2014-2016 [16]. There was limited evidence on survival among people initiating HAART in Ethiopia as of 2017. Our study revealed mortality in the year 2017-2019 to be 9.25% which was similar with a study conducted in Nepal that reported 6.3% mortality during the 36 months of follow-up [35]. The different reports in mortality may be explained by the studies that were specific to limited number of health facilities while mortality is heterogeneous across the different health facilities and by duration on HAART.

Despite previous studies [17, 34, 36–38] revealed that mortality was the highest in the first year of follow-up, our study showed that mortality was increasing overtime during the 2007-2009 and 2010-2013 cohorts. This might be explained by the challenges of adherence to medication, patients' retention in care, and the limited access to HAART in Ethiopia during the year 2007-2013 [12, 21, 39, 40]. The year 2014-2016 was the time with the minimum mortality in the first year of follow-up (5.14%) across all the cohorts, and mortality was steadily declining until 2016 (i.e. 4.04% and 4.43%) which may explain the revised national program on the test and treat strategy as of 2014 and the overtime increased access to HAART [41]. However, there were high rates of mortality in the first year of follow-up during the year 2017 to 2019 (18.76%) which might be explained by the political instability in the country with negative impacts on the health service from 2017 to 2018. This negative impact could be proofed by the greatly reduced rate of 1.35% for good and fair adherence to HAART in cohort initiating HAART by 2017, compared to that of 98.8% to 99.7% in rest of the cohorts. Our study also revealed variations in mortality among the different regional administrations in Ethiopia which could be explained by the different sociocultural factors and access to health service. Moreover, the heterogeneity of HIV prevalence among the regions could contribute to the variation of early mortality.

Between 2007 and 2016, survival of people living with HIV in the first 3 years since ART initiation improved substantially. However, there was a decline in survival as of 2017 to 2019. Overall survival among PLHIV initiating HAART in Ethiopia from 2007 to 2019 within three years of follow-up was 78.8% (95%CI = 61.39 − 95.17). This study was consistent with a study conducted in Debre Berhan Hospital, Ethiopia, which reported 81.7% survival during 2017 [18]. Survival among the cohorts of 2007-2009, 2010-2013, 2014-2016, and 2017-2019 was heterogeneous, i.e., 53.71%, 79.51%, 92.54%, and 87.36%, respectively. Briefly, the lowest survival during 2007-2009 may explain the limited access to HAART by the time and also issues of LTFU and adherence during these cohorts [42–44]. The steady improvement of survival during the 2010-2013 and 2014-2016 might be explained by the different program implementations in the country overtime, specifically the test and treat strategy and rapid expansion of ART sites [41]. However, survival among people initiating HAART as of 2017 is declining which might be explained by the challenges related to instability and supply sustainability in the country.

The high mortality among bedridden patients (22.01%) during the year 2007-2010 may be explained by the late diagnosis of PLHIV as a result of the limited access to HIV testing, high rate stigma, and the limited access to HAART by the time [45, 46]. Bedridden patients were at 2.0 (95%CI = 1.35 − 2.69) times risk of death compared to ambulatory patients. Being bedridden was also predictor of mortality during the 2007-2010 and 2011-2013 follow-ups that accounted 1.92 (95%CI = 1.28, 2.89) and 2.30 (95%CI = 1.40, 3.78) times risk of mortality, respectively. This was consistent with a study in Nepal which reported 2 twofold risk of death among bedridden compared to ambulatory patients [35]. Other study conducted in Debre Berhan Hospital, Ethiopia, also revealed a 3-fold risk of death among bedridden [18]. Other study conducted in Ethiopia also revealed being bedridden as 17.4 times risk of death compared to ambulatory patients [34]. A study conducted in Gonder, Ethiopia, also revealed a 9.57 times risk of death among bedridden [8]. Our study was consistent with other previous studies and may highlight the importance of early HIV testing and treatment to ensure better survival of PLHIV.

Mortality among patients with poor adherence was as high as 90.91%, 84.21%, and 50.00% for cohorts initiating HAART by 2007, 2010, and 2014, respectively, but not as high as 9.2% for cohort initiating HAART by 2017. Moreover, patients with poor adherence in all cohorts were at 6.78 (95%CI = 3.4 − 10.3) times risk of death compared to those who had good adherence. Patients with poor adherence were at 4.65 (95%CI = 2.90, 7.48), 12.29 (95%CI = 7.06, 21.40), 8.54 (95%CI = 2.71, 26.94), and 7.07 (95%CI = 3.74, 13.38) times risk of mortality for all the cohorts, respectively, compared to those with good adherence. Former studies are also consistent with our report which was conducted in Gamo Gofa Zone, Ethiopia, that reported a 2.17 times risk of death compared to those with good adherence [16]. The current study highlights the importance of improving adherence among people initiating HAART which could be a critical challenge at the early stage of HAART initiation. Adherence to ART in HIV-infected individuals is reported to be strong determinant of disease outcome [47]. Interventions which improve adherence are associated with successful viral suppression, reduced risk of opportunistic infections, and prevention of drug resistance [5]. Although adherence levels as low as 80% have been associated with treatment success, adherence of around 95% is widely considered desirable for viral suppression and prevention of ART resistance [48].

There was high mortality among patients with CD4 count ≤ 200 cells/mm^3^ across all the different segments of the cohorts which accounted 25.73%, 12.55%, 5.65%, and 18.65% during the 2007-2010, 2011-2013, 2014-2016, and 2017-2019, respectively. Patients with CD4 count of ≤200 cells/mm^3^ were at 2.6 (95%CI = 2.2 − 3.0) times risk of death compared to those with CD4 count greater than 200 cells/mm^3^. While disaggregated by the different cohorts, patients with CD4 count of ≤200 cells/mm^3^ were at 2.71 (95%CI = 2.01, 3.66), 2.57 (95%CI = 1.93, 3.42), 2.46 (95%CI = 1.60, 3.77), and 2.70 (95%CI = 2.0, 3.64) during 2007-2010, 2011-2013, 2014-2016, and 2017-2019, respectively, compared to those with CD4 count > 200 cells/mm^3^. Studies showed that CD4 count is one of the predictors of mortality in the sense that higher CD4 counts are related to longer survival time [8, 16, 26, 34, 38, 49, 50]. A study conducted in Jimma University teaching hospital, Ethiopia, indicated that patients who were on ART with CD4 < 200 cells/mm^3^ were 1.3 times more likely to die in comparison to those whose CD4 was ≥200 cells/mm^3^. The reduction of CD4 count decreases the immunity of patients, exposing them to opportunistic infections and at high risk of developing serious illnesses leading to death [9, 18, 27, 51].

In our analysis, patients who were not virally suppressed at baseline were at 4.66 times risk of death and hence underscore the importance of viral load measurement as the primary marker of treatment efficacy and suggest the importance of switching ART regimens when there is evidence of virologic failure. Previous study conducted in China also demonstrated an association between HIVDR and mortality, consistent with previous studies. In addition, patients who developed HIVDR during treatment were nearly twofold more likely to die than those who developed HIVDR later [47]. Our study also revealed that HIV drug resistance was found to be a 10-fold risk of mortality compared to those who were susceptible.

The highest LTFU during 2007-2010 in our study (25.26%) was consistent with previous study conducted in 2010 which was 23.21% [52]. Moreover, the high rate of LTFU by the year 2017-2019 which accounted 19.72% may be explained by the political instability during this period which impacted on health service in the country. High rate of LTFU could lead towards HIV drug resistance and also high rate of community HIV transmission ultimately affects the prevention and control strategy.

## 5. Limitation

There might be death among those groups of LTFU population which may underestimate the rate of mortality in this study.

## 6. Conclusion

Even in the late HAART era, survival during the first 3 years of HAART continues to improve until 2016. However, it was significantly declining as of 2017. This calls for close follow-up and monitoring of PLHIV taking HAART. Poor adherence, low baseline CD4 count, and bedridden were predictors of mortality which calls for attention during clinical management of this group of population. Moreover, clinicians should consider viral load suppression at baseline since it is impacting survival. The high rate of loss to follow-up in recent years requires program attention.

## Figures and Tables

**Table 1:** Demographic characteristics of people taking HAART in Ethiopia (2007-2019) enrolled to the study (*N* = 11,013).

Variable		Frequency	Proportion (%)
Region	Tigray	1504	13.66
	Afar	623	5.66
	Amhara	1555	14.12
	Oromia	1542	14
	Somalia	448	4.07
	Benishangul	487	4.42
	SNNP	1391	12.63
	Gambella	678	6.16
	Harari	610	5.54
	Dire Dawa	452	4.1
	Addis Ababa	1723	15.65

Gender	Female	7152	64.94
	Male	3861	35.06

Residency	Urban	10089	91.61
	Rural	924	8.39

Educational status	No formal education	2764	25.1
	Primary school education	4720	42.86
	Secondary/high school education	2174	19.74
	College or university	1355	12.3

Marital status	Single	1306	11.86
	Married	5381	48.86
	Divorced	2261	20.53
	Widowed	2065	18.75

Occupation	Government employees	2087	18.95
	Merchant	1767	16.04
	Driver	335	3.04
	Housewives	1840	16.71
	Student	386	3.5
	Unemployed	1435	13.03
	Other	3163	28.72

Age (year)	15-24	5	0.05
	25-34	436	3.96
	35-44	2870	26.06
	45-54	4314	39.17
	55-64	2244	20.38
	65-74	877	7.96
	75-84	235	2.13
	≥85	32	0.29

Total		11,013	

**Table 2:** Mortality among PLHIV initiating HAART in Ethiopia disaggregated by different characteristics and calendar years of HAART initiation (2007-2019).

Characteristics		2007-2010	2011-2013	2014-2016	2017-2019
Region	Tigray	66/307 (21.50)	53/567 (9.35)	16/374 (4.28)	30/256 (11.72)
	Afar	11/58 (18.97)	19/220 (8.64)	10/187 (5.35)	11/158 (6.96)
	Amhara	69/282 (24.47)	56/554 (10.11)	27/414 (6.52)	22/305 (7.21)
	Oromia	46/210 (21.90)	48/538 (8.92)	19/454 (4.19)	31/340 (9.12)
	Somalia	10/49 (20.41)	14/136 (10.29)	11/144 (7.64)	16/119 (13.45)
	Benishangul	23/120 (19.17)	17/155 (10.97)	4/126 (3.17)	5/86 (5.81)
	SNNP	29/191 (15.18)	57/542 (10.52)	12/391 (3.07)	22/267 (8.24)
	Gambella	19/76 (25.00)	13/208 (6.25)	7/192 (3.65)	10/202 (4.95)
	Harari	23/113 (20.35)	18/182 (9.89)	5/172 (2.91)	21/143 (14.69)
	Dire Dawa	9/51 (17.65)	21/165 (12.73)	4/154 (2.60)	12/82 (14.63)
	Addis Ababa	97/424 (22.88)	72/601 (11.98)	18/396 (4.55)	29/302 (9.60)

Age (year)	15-24	5/37 (13.51)	5/98 (5.10)	2/130 (1.54)	15/171 (8.77)
	25-34	45/254 (17.72)	89/849 (10.48)	39/931 (4.19)	66/835 (7.90)
	35-44	165/783 (21.07)	150/1578 (9.51)	56/1175 (4.77)	82/779 (10.53)
	45-54	122/523 (23.33)	92/887 (10.37)	22/534 (4.12)	25/300 (8.33)
	55-64	44/208 (21.15)	43/351 (12.25)	10/183 (5.46)	16/135 (11.85)
	≥65	21/76 (27.63)	9/105 (8.57)	4/51 (7.84)	5/40 (12.50)

Gender	Female	244/1168 (20.89)	251/2490 (10.08)	80/1987 (4.03)	131/1507 (8.69)
	Male	158/713 (22.16)	137/1378 (9.94)	53/1017 (5.21)	78/753 (10.36)

Residency	Urban	383/1797 (21.31)	350/3575 (9.79)	118/2683 (4.40)	188/2034 (9.24)
	Rural	19/84 (22.62)	38/293 (12.97)	15/321 (4.67)	21/226 (9.29)

Educational status	No formal education	78/378 (20.63)	81/958 (8.46)	45/786 (5.73)	60/642 (9.35)
	Primary school education	159/778 (20.44)	183/1660 (11.02)	51/1323 (3.85)	72/959 (7.51)
	Secondary school	105/448 (23.44)	70/746 (9.38)	25/575 (4.35)	44/405 (10.86)
	College or university	60/277 (21.66)	54/504 (10.71)	12/320 (3.75)	33/254 (12.99)

Marital status	Single	35/181 (19.34)	44/411 (10.71)	17/369 (4.61)	37/345 (10.72)
	Married	186/886 (20.99)	180/1920 (9.38)	67/1469 (4.56)	103/1106 (9.31)
	Divorced	88/381 (23.10)	76/740 (10.27)	28/665 (4.21)	43/475 (9.05)
	Widowed	93/433 (21.48)	88/797 (11.04)	21/501 (4.19)	26/334 (7.78)

Occupation	Government employee	88/405 (21.73)	88/805 (10.93)	18/526 (3.42)	35/351 (9.97)
	Merchant	65/289 (22.49)	63/632 (9.97)	25/489 (5.11)	37/357 (10.36)
	Driver	23/77 (29.87)	12/108 (11.11)	7/91 (7.69)	7/59 (11.86)
	Housewife	67/295 (22.71)	66/648 (10.19)	18/479 (3.76)	38/418 (9.09)
	Student	9/57 (15.79)	14/122 (11.48)	1/116 (0.86)	12/91 (13.19)
	Unemployed	39/223 (17.49)	50/489 (10.22)	21/424 (4.95)	23/299 (7.69)
	Other	111/535 (20.75)	95/1064 (8.93)	43/879 (4.89)	57/685 (8.32)

Type of HAART	ABC, 3TC, EFV	1/6 (16.67)	1/11 (9.09)	0/6 (0.00)	0/4 (0.00)
	AZT, 3TC, EFV	41/192 (21.35)	51/417 (12.23)	10/225 (4.44)	14/80 (17.50)
	ABC, 3TC, NVP	97/438 (22.15)	109/1035 (10.53)	17/448 (3.79)	9/102 (8.82)
	AZT, 3TC, NVP	1/2 (50.00)	0/4 (0.00)	3/79 (3.80)	118/1278 (9.23)
	TDF, 3TC, EFV	133/608 (21.88)	110/1040 (10.58)	55/1184 (4.65)	141/1464 (9.63)

Functional status-baseline	Ambulatory	372/1690 (15.71)	368/3649 (10.08)	127/2937 (4.32)	11/73 (15.07)
	Bedridden	30/191 (22.01)	20/219 (9.13)	6/67 (8.96)	209/2260 (9.25)

WHO clinical stage	Clinical stage I	14/73 (19.18)	32/423 (7.57)	23/653 (3.52)	42/559 (7.51)
	Clinical stage II	68/330 (20.61)	65/813 (8.00)	36/774 (4.65)	89/747 (11.91)
	Clinical stage III	238/1149 (20.71)	246/2161 (11.38)	60/1341 (4.47)	17/147 (11.56)
	Clinical stage IV	82/329 (24.92)	45/471 (9.55)	14/236 (5.93)	209/2260 (9.25)

^∗∗^Adherence	Good	370/1842 (20.09)	366/3829 (9.56)	128/2983 (4.29)	0/14 (0.00)
	Fair	12/17 (70.59)	6/20 (30.00)	0/11 (0.00)	4/17 (719.25)
	Poor	20/22 (90.91)	16/19 (84.21)	5/10 (50.00)	209/2260 (64.01)

CD4 count (cells/mm^3^)	≤200	334/1298 (25.73)	317/2526 (12.55)	95/1680 (5.65)	175/1600 (18.65)
	>201	49/514 (9.53)	57/1237 (4.61)	27/1265 (5.65)	125/821 (15.23)

Hemoglobin (mg/dl)	≤12.5	182/892 (20.40)	162/1333 (12.15)	33/744 (4.45)	392/1961 (19.98)
	>12.5	217/984 (22.05)	226/2525 (8.95)	100/2252 (4.45)	31/299 (10.37)

Viral suppression	Yes	334/1298 (25.73)	317/2526 (12.55)	95/1680 (5.65)	175/1600 (18.65)
	No	49/514 (9.53)	57/1237 (4.61)	27/1265 (5.65)	125/821 (15.23)

^∗^HIV-DR	Yes	544/699 (77.8)			
	No	155/699 (22.2)			

Total		402/1881 (21.37)	388/3868 (10.03)	133/3004 (4.43)	209/2260 (9.25)

Lost to follow-up (LTFU)		500/2283 (25.26)	708/4479 (16.90)	204/3196 (6.63)	504/2685 (19.72)

^∗^HIV drug resistance testing was done regardless of the duration on treatment for patients with two consecutive VL > 1000 copies/ml. The details of this HIVDR and viral load suppression report are published [53, 54].

**Table 3:** Predictors of mortality within the first three of HAART initiation among PLHIV in Ethiopia (2007-2019).

Characteristics	2007-2010 (CHR)		2007-2010 (aHR)	2011-2013(CHR)		2011-2013 (aHR)		2014-2016(CHR)	2014-2016 (aHR)	2017-2019 (CHR)		2017-2019 (aHR) pooled aHR
	CHR	(95.0% CI), sig.	aHR (95% CI),sig.	CHR	(95.0% CI), sig.	aHR (95.0% CI0, sig.	CHR	(95.0% CI), sig.	aHR (95% CI),sig.	CHR	(95.0% CI), sig.	aHR (95.0% CI) sig. aHR (95.0% CI)
*Region*												
Tigray (ref.)	1											
Afar	0.9	(0.64, 1.25) 0.52		0.81	(0.56, 1.17) 0.26		0.9	(0.45, 1.79) 0.75		1.28	(0.75, 2.17) 0.36	1.69 (0.99, 2.89) 0.05
Amhara	1.21	(0.64, 2.31) 0.56		0.81	(0.48, 1.37) 0.44		1.13	(0.51, 2.51) 0.76		0.82	(0.40, 1.68) 0.58	0.99 (0.47, 2.09) 0.98
Oromia	1.24	(0.90, 1.71) 0.19		0.83	(0.58, 1.19) 0.30		1.43	(0.77, 2.65) 0.26		0.73	(0.41, 1.30) 0.29	0.94 (0.53, 1.68) 0.84
Somalia	1.14	(0.79, 1.64) 0.48		0.74	(0.51, 1.07) 0.11		0.92	(0.48, 1.77) 0.80		0.96	(0.57, 1.60) 0.86	1.3 (0.76, 2.23) 0.34
Benishangul	0.97	(0.50, 1.87) 0.92		0.85	(0.48, 1.52) 0.58		1.57	(0.73, 3.36) 0.25		1.55	(0.83, 2.90) 0.17	1.66 (0.89, 3.12) 0.11
SNNP	0.94	(0.58, 1.51) 0.80		0.8	(0.46, 1.38) 0.42		0.79	(0.26, 2.36) 0.67		0.58	(0.22, 1.51) 0.26	0.64 (0.24, 1.69) 0.37
Gambella	0.78	(0.51, 1.20) 0.26		0.97	(0.68, 1.38) 0.85		0.66	(0.31, 1.40) 0.28		0.96	(0.54, 1.69) 0.88	1.31 (0.72, 2.41) 0.38
Harari	1.28	(0.78, 2.12) 0.33		0.58	(0.32, 1.05) 0.07		0.84	(0.34, 2.05) 0.70		0.57	(0.28, 1.20) 0.14	0.86 (0.41, 1.8) 0.69
Dire Dawa	1.05	(0.66, 1.66) 0.85		0.71	(0.42, 1.20) 0.20		0.66	(0.24, 1.80) 0.42		1.59	(0.90, 2.81) 0.11	2.01 (1.1, 3.66) 0.02
Addis Ababa	1.1	(0.55, 2.20) 0.78		0.96	(0.59, 1.58) 0.88		0.59	(0.20, 1.75) 0.34		1.65	(0.84, 3.28) 0.15	1.94 (0.95, 3.96) 0.07
*Education*												
No education	1.15	(0.81, 1.62) 0.44		0.86	(0.59, 1.25) 0.43	0.91 (0.63, 1.32) 0.63	1.02	(0.49, 2.16) 0.95		0.81	(0.50 ,1.33) 0.41	0.83 (0.52, 1.35) 0.46
Primary school	1.16	(0.78, 1.74) 0.46		0.79	(0.53, 1.20) 0.27	0.82 (0.58, 1.18) 0.29	1.37	(0.64, 2.92) 0.41		0.73	(0.44, 1.24) 0.24	0.81 (0.52, 1.26) 0.35
Secondary school	1.08	(0.77, 1.52) 0.66		1.08	(0.76, 1.52) 0.68	1.07 (0.78, 1.47) 0.66	0.94	(0.46, 1.91) 0.87		0.54	(0.34, 0.87) 0.01	0.58 (0.38, 0.9) 0.01
College (ref.)	1											
*Functional status*												
Ambulatory (ref.)	1											
Bedridden	1.69	(1.05 ,2.75) 0.03	1.92 (1.28, 2.89) 0.00	2.06	(1.15, 3.68) 0.01	2.30(1.40, 3.78) 0.00	1.1	(0.31, 2.61) 0.95		0.96	(0.44, 2.13) 0.93	^∞^2.0 (1.35-2.69)
Adherence												
Good (ref.)	1											
Fair	0.18	(0.10, 0.35) 0.00	3.77 (1.95, 7.32) 0.00	0.07	(0.03, 0.14) 0.00	3.10 (1.37, 7.05) 0.01	0.14	(0.02, 1.05) 0.06	0.00 (0.00, 0.001) 0.95	0.14	(0.06, 0.30) 0.00	0 (0, 0.001) 0.94
Poor	7.90	(2.26, 12.37) 0.01	4.65 (2.90, 7.48) 0.00	12.25	(10.07, 15.93) 0.04	12.29 (7.06, 21.40)0.00	6.46	(2.00, 11.03) 0.02	8.54 (2.71, 26.94) 0.00	5.42	(4.00, 9.01) 0.03	7.07 (3.74, 13.38) 0.01 ^∞^6.78 (3.4-10.3)
*CD4 count (cells/mm* ^3^)												
≤200	2.67	(1.83, 3.90) 0.00	2.71 (2.01, 3.66) 0.00	2.38	(1.69, 3.36) 0.00	2.57 (1.93, 3.42) 0.00	2.68	(1.57, 4.59) 0.00	2.46 (1.60, 3.77) 0.00	2.74	(1.87, 4.00) 0.00	2.7 (2.0, 3.64) 0.01 ^∞^2.6 (2.2-3.0)
>201 (ref.)	1											
*HIVDR*												
Yes	10.2	(4.31, 18.4) 0.03	9.42 (12.52, 6.64) 0.021	9.21	(4.31, 14.62) 0.01	8.29 (5.81, 12.42) 0.002	9.67	(5.44, 13.01) 0.00	11.01 (7.22, 15.81) 0.002	10.62	(7.51, 13.74) 0.01	10.31 (7.41, 13.92) 0.001 ^∞^10.02 (6.91, 13.82)
No (ref.)	1											
*Viral suppression*												
Suppressed (ref.)	1											
Not suppressed	4.21	(2.37, 6.41) 0.00	3.92 (1.90, 5.63) 0.00	4.62	(2.51, 6.32) 0.00	5.32 (2.61, 8.22) 0.01	3.22	(1.37, 5.68) 0.00	5.26 (2.54, 7.62) 0.02	4.41	(2.36, 6.72)	3.81 (1.76, 5.51), 0.00 ∞4.66 (2.53,6.72)

Key: variables with *P* value > 0.2 for CHR during either of the cohorts were excluded from the table. ^∗^Ref.: reference categories were selected as the least risk groups according to the different literatures [8, 9, 36, 49, 50, 53]. ^∞^Pooled estimate of aHR (i.e., for the cohorts initiating HAART by 2007 and 2010 for bedridden. We considered all cohorts for CD4 count ≤ 200 cells/mm^3^ and poor adherence.

## Data Availability

The raw data can be obtained from the corresponding author upon reasonable formal request.

## Abbreviations

AIDS: Acquired immune deficiency syndrome
ART: Antiretroviral therapy
CI: Confidence interval
EPHI: Ethiopian Public Health Institute
HAART: Highly active antiretroviral therapy
HGB: Hemoglobin
HIV: Human immunodeficiency virus
HIVDR: HIV drug resistance
HR: Hazard ratio
Mg/dl: Milligram per deciliter
ZJU: Zhejiang University.
